# Enterovirus 71 Suppresses miR-17-92 Cluster Through Up-Regulating Methylation of the miRNA Promoter

**DOI:** 10.3389/fmicb.2019.00625

**Published:** 2019-03-28

**Authors:** Yuxuan Fu, Li Zhang, Rui Zhang, Shijie Xu, Huanru Wang, Yu Jin, Zhiwei Wu

**Affiliations:** ^1^School of Life Sciences, Ningxia University, Yinchuan, China; ^2^Center for Public Health Research, Medical School of Nanjing University, Nanjing, China; ^3^Jiangsu Key Laboratory of Infection and Immunity, Institutes of Biology and Medical Sciences, Soochow University, Suzhou, China; ^4^Nanjing Children’s Hospital, Nanjing Medical University, Nanjing, China; ^5^The State Key Laboratory of Analytical Chemistry for Life Sciences, Nanjing University, Nanjing, China; ^6^Jiangsu Key Laboratory of Molecular Medicine, Medical School, Nanjing University, Nanjing, China

**Keywords:** enterovirus 71, hsa-miR-17∼92, promoter, methylation, DNMT3B

## Abstract

Enterovirus 71 (EV71), the etiological agent of hand-foot-and-mouth disease, has become an increasing public health challenge worldwide. Accumulating evidence suggests that mammalian microRNAs (miRNAs), a class of non-coding RNAs of 18 to 24 nucleotides (nt) with important regulatory roles in cellular processes, participate in host antiviral defense and studies have suggested roles of miRNAs in EV71 replication and pathogenesis. In the current study, we reported that the expression of hsa-miR-17∼92 cluster was significantly downregulated during EV71 infection. Overexpression of hsa-miR-17∼92 inhibited, while inhibition of endogenous hsa-miR-17∼92 facilitated EV71 replication. We identified two sequences located at nt 3024 to 3038 and nt 2838 to 2862 of the EV71 (strain FY0805) genome as potential targets for hsa-miR-17-5p and miR-19a/b, respectively, which were validated by luciferase reporter assays and Western blot. Meanwhile, we identified DNA methylation as a novel mechanism of hsa-miR-17∼92 regulatory roles. The methylation of the miR-17-92 promoter was significantly increased (50%) upon EV71 infection, which appeared to be caused by the increased expression of DNMT3B but not DNMT1 and DNMT3A. Furthermore, we demonstrated that the members of miR-17-92 cluster were decreased in the sera of EV71 infected patients, suggesting the clinical implication and the potential therapeutic application of miR-17-92.

## Introduction

Enterovirus 71 (EV71), a small, non-enveloped, icosahedral RNA virus that belongs to the family *Picornaviridae* and an etiological agent for hand-foot and-mouth disease (HFMD), has caused several outbreaks worldwide and sometimes resulted in severe neurological disorders and mortality in children (McMinn, 2002; Solomon et al., 2010; Chang et al., 2016). Although a vaccine is available recently (Mao et al., 2016), its clinical efficacy is not yet known. At present, the pathogenesis of EV71 is still poorly understood and antiviral therapies are limited. Therefore, it is imperative to identify potential antiviral targets or potential antiviral agents.

Virus infections can alter host gene expression, including miRNAs, contributing to viral propagation and pathogenesis (Skalsky and Cullen, 2010). MicroRNAs (miRNAs) are abundant small non-coding RNAs (ncRNAs), ∼19–24 nucleotide nucleotides (nts) in length, and play crucial roles in regulating both cellular and viral gene expression (Gottwein and Cullen, 2008; Lee and Dutta, 2009). The expression of host miRNAs during EV71 infection has been the focus of much interest. Accumulating evidence suggests that human miRNAs, such as miR-30a, miR-146a, and miR-127-5p, control EV71 infection or replication (Ho et al., 2014; Fu et al., 2015; Feng et al., 2017). Our previous study also showed that EV71 repressed the has-miR-30a expression to induce autophagic activity and benefit its replication (Fu et al., 2015). Those findings suggested that cellular miRNA-virus interaction may serve as a novel regulatory mechanism for antiviral therapy.

The miR-17-92 cluster encodes six individual mature miRNAs, including miR-17, miR-18a, miR-19a, miR-20a, miR-19b-1, and miR-92a. A number of studies have reported that endogenous miR-17-92 cluster could either directly interact with human viral genomes or indirectly effect on viral replication. Yang et al. explored the endogenous miRNA-17-92 cluster to generate five mature miRNAs that targeted different regions of the HCV genome to inhibit viral replication, which provided a promising candidate for the treatment of HCV infection (Jung et al., 2013). Another report showed that miR-17-92 did not directly target the HIV-1 viral genome instead affected virus replication by targeting histone acetylase PCAF, which is an important cofactor for Tat in HIV-1 gene expression (Triboulet et al., 2007).

In the current study, the expression profiles of cellular miRNAs in infected and non-infected cells were compared by microarray analysis and we observed that miR-17-92, one of the best characterized polycistronic miRNA clusters (Mendell, 2008; Olive et al., 2013a), was substantially reduced upon EV71 infection and overexpression of this cluster inhibited viral replication. Further studies using a luciferase reporter found that miR-17, miR-19a, and miR-19b could bind the EV71 genome. Mechanistic studies showed that the methylation of the miR-17-92 promoter was significantly increased upon EV71 infection, which appeared to be caused by the increased expression of DNMT3B. Furthermore, we demonstrated that the members of miR-17-92 cluster were reduced in the sera of EV71 infected patients, and the reduction was correlated with disease severity, suggesting the clinical relevance of miR-17-92 cluster.

## Results

### The miR-17∼92 Cluster Was Down-Regulated During EV71 Infection

**FIGURE 1:**
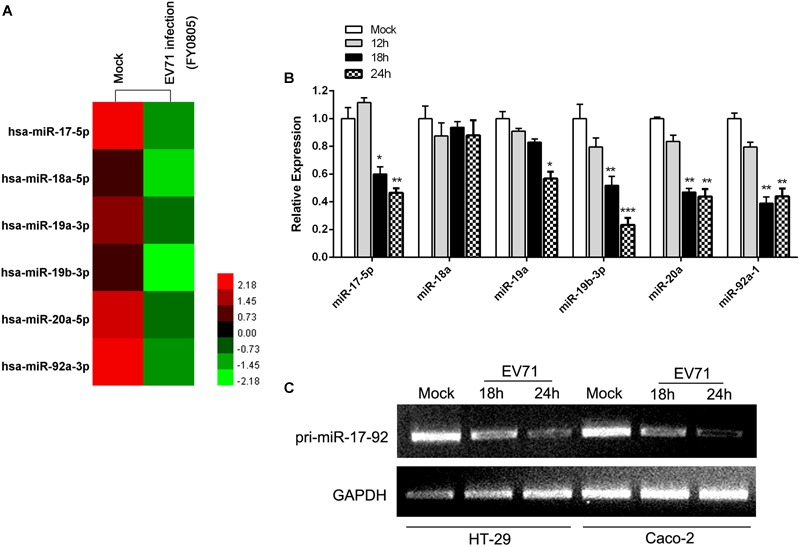
EV71 infection downregulated miR-17-92 cluster. **(A)** The heatmap showed the expression level of members of miR-17-92 cluster in mock- and EV71-infected HT-29 cells by small RNA deep sequencing. **(B)** Expression of mature miRNAs of the miR-17-92 cluster was analyzed in EV71-infected HT-29 cells at indicated time points by real-time quantitative PCR, using U6 rRNA as an internal control. Values were mean of triplicate experiments and presented in relative to the level of the mock infection. Mock infection was assigned with a value of 1. The data were presented as mean ± SEM (*n* = 3, ^∗^*P* < 0.05, ^∗∗^*P* < 0.01, ^∗∗∗^*P* < 0.001). **(C)** RT-PCR analysis of the pri-miR-17-92 cluster expression in EV71-infected cells at indicated time points normalized against GAPDH transcript levels.

To investigate the impact of EV71 infection on the cellular miRNAs, we performed small RNA deep sequencing analysis on host cellular miRNAs in HT-29 cells infected with EV71 at a MOI of 1 for 24 h. On the basis of differentially expressed miRNAs, we identified 101 miRNAs that were modulated by EV71 infection (Supplementary Figures S1A,B). Among these miRNAs, six miRNAs, miR-17, miR-18a, miR-19a, miR-20a, miR-19b-1, and miR-92a, encoded by polycistronic miR-17-92 cluster, were all substantially reduced upon EV71 infection (Figure 1A). Expression of mature miRNAs of the miR-17-92 cluster was confirmed by real-time quantitative PCR. All miRNAs except the miR-18a, were downregulated by EV71 replication (Figure 1B). Moreover, RT-PCR analysis also confirmed that the pri-miR-17-92 cluster expression was significantly reduced, suggesting that EV71 infection suppresses transcription of the miR-17-92 cluster (Figure 1C).

### The Expression Level of miR-17-92 Cluster Affected EV71 Replication

**FIGURE 2:**
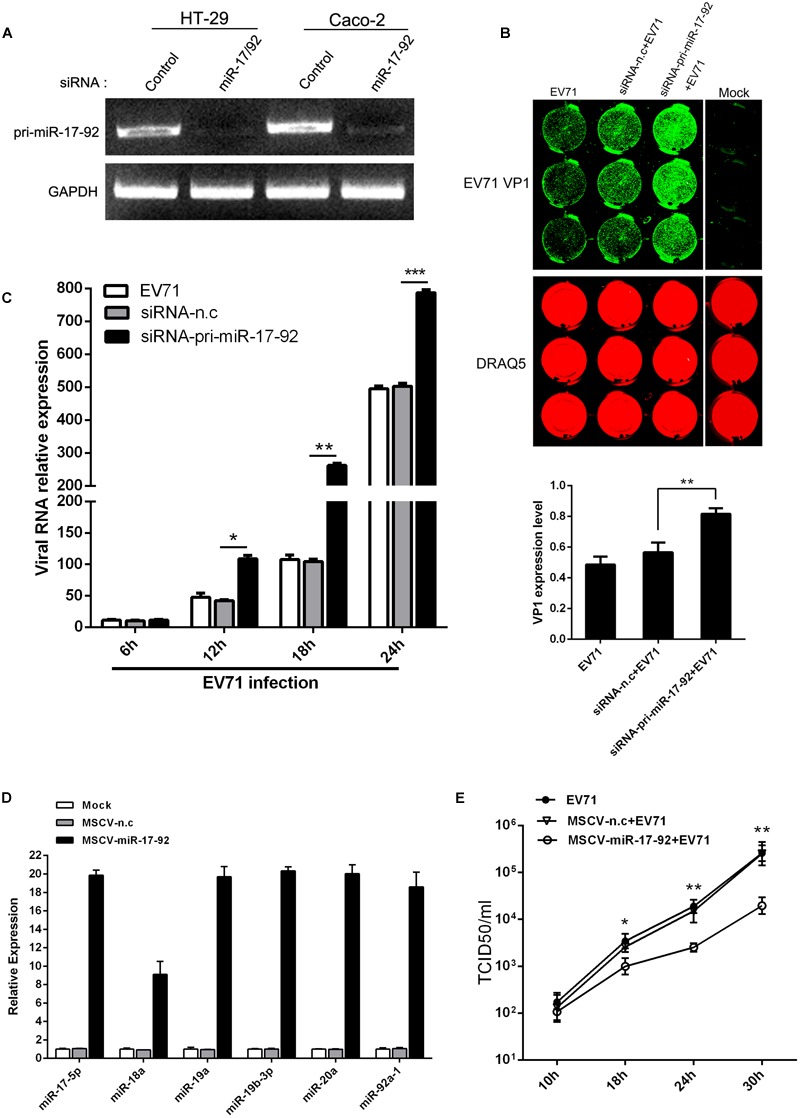
The expression level of miR-17-92 cluster affected EV71 replication. **(A)** RT-PCR analysis of pri-miR-17-92 cluster expression after HT-29 or Caco-2 cells were transfected with si-pri-mir-17-92 for 48 h. **(B, C)** HT-29 cells were infected with EV71 (MOI = 1) prior to being transfected with si-pri-mir-17-92 for 24 h. EV71 VP1 expression level was determined via In-cell Western and normalized by DRAQ5 **(B)**, and viral RNA level was quantified via real-time quantitative PCR at indicated time points normalized against GAPDH transcript level **(C)**. **(D)** Quantitative real-time-PCR analysis of miR-17-92 component expression in HT-29 cells when the cells were transfected with MSCV-miR-17-92 vector or negative control (MSCV-n.c) for 48 h. **(E)** Titers of progeny EV71 in the culture supernatant of infected-cells before the cells were transfected with MSCV-miR-17-92 vector or negative control. All experiments were performed three times and the representative results were shown. The data were presented as mean ± SEM (^∗^*P* < 0.05, ^∗∗^*P* < 0.01, ^∗∗∗^*P* < 0.001).

To investigate the effect of miR-17-92 expression on EV71 replication, HT-29 and Caco-2 cells were transfected with chemically synthesized siRNAs to evaluate the effect of the cluster downregulation on viral replication (Figure 2A). The results showed that knockdown of pri-miR-17-92 with specific siRNA enhanced EV71 replication in these two cell lines as determined by In-Cell Western assay (Figure 2B) and real-time quantitative PCR (Figure 2C), suggesting that endogenous miR-17-92 is a restriction factor for viral replication. Meanwhile, transfection of MSCV-miR-17-92 into the HT-29 cells, which expresses intact miR-17-92 components (Olive et al., 2013b), significantly inhibited extracellular EV71 titers (Figure 2E) as the cells overexpressed members of miR-17-92 cluster at 9.1- to 20.3-fold of the negative control (Figure 2D). These observations suggest that this miRNA cluster inhibits the viral replication.

### The miR-17-5p, miR-19a and miR-19b Directly Targeted the EV71 Genome

**FIGURE 3:**
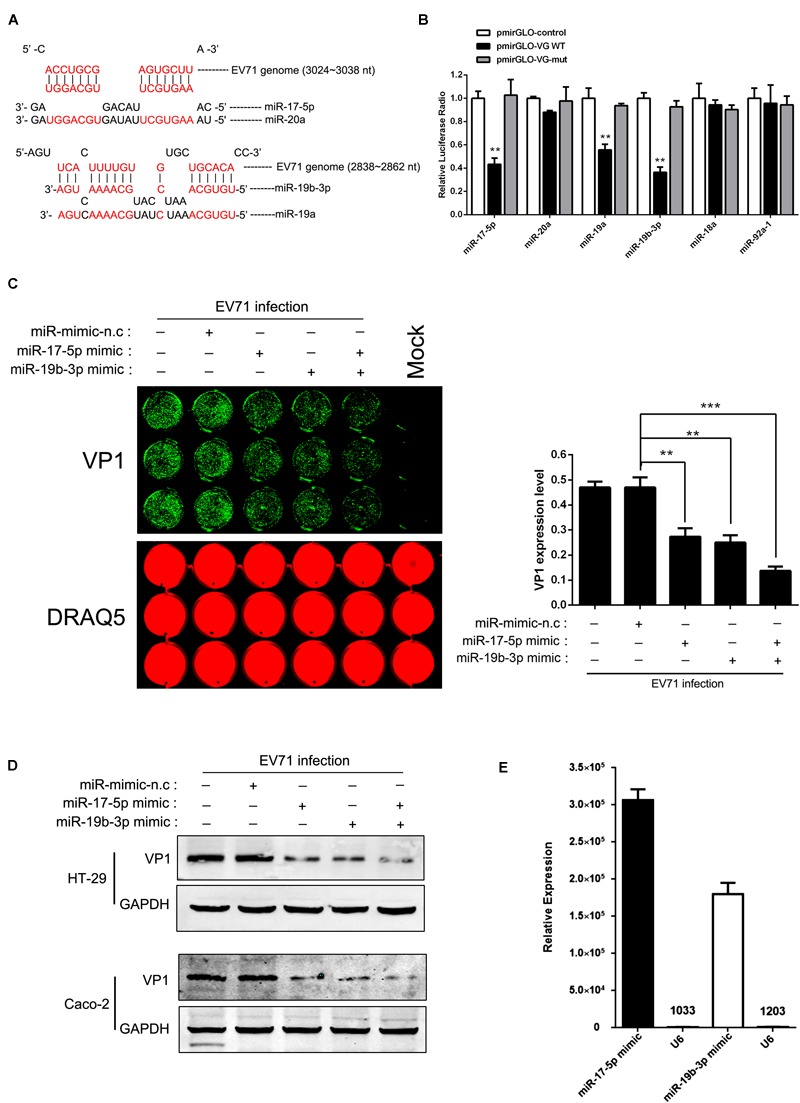
miR-17-5p, miR-19a, and miR-19b directly targeted the EV71 genome. **(A)** Potential target sites of mature miRNAs of the miR-17-92 cluster in the EV71 genome. Computational analysis with RNA-hybrid software yielded potential binding sites for miR-17, miR-20a, and miR-19a/b in the EV71 genome. **(B)** EV71 genome containing presumptive miR-17-92 target sequences, was cloned into the multiple cloning sites at the 3′ of the firefly luciferase reporter gene (luc2) in the luciferase expression plasmid (pmirGLO). Either control plasmid (pmirGLO-control), pmirGLO-viral genome WT (pmirGLO-VG WT) or pmirGLO-viral genome mut (pmirGLO-VG-m) was co-transfected with individual miRNA mimic in HT-29 cells. The luciferase activity of pmirGLO-VG WT group was repressed by transfecting miR-17 and miR-19a/b while mutation of a predicted miRNAs binding sites abolished miRNAs-mediated repression. **(C,D)** HT-29 cells and Caco-2 were infected with EV71 (MOI = 1) after being transfected with miR-17 or miR-19b mimic for 24 h. EV71 VP1 expression level was determined via In-cell Western and normalized by DRAQ5 **(C)** or Western blot **(D)**. The symbols “+” and “-” indicate treatment and no treatment, respectively, by the corresponding factor. **(E)** HT-29 cells transfected with miR-17 or miR-19b mimic were subjected to Ago2-IP, and copy numbers of miR-17 and miR-19b in the immunoprecipitates were determined by real-time quantitative PCR, using the U6 as a negative control. All the results shown were representative of three independent experiments and data were presented as mean ± SEM (^∗∗^*P* < 0.01, ^∗∗∗^*P* < 0.001).

We used a bioinformatic program to predict the target sites of the miR-17-92 cluster in the EV71 genome (RNA-hybrid and PITA predictions) and identified that the structural protein coding region contains two putative miR-17-5p/miR-20a and miR-19a/miR-19b binding sites (nt 3024 to 3038 for miR-17-5p/miR-20a and nt 2838 to 2862 for miR-19a/miR-19b, respectively; Figure 3A). The EV71 genomic fragments containing the putative binding sites were cloned into the pmirGLO miRNA target expression vector for the analysis of the direct interaction between these four miRNAs and EV71 genome by luciferase reporter assay. Mutated target sequences were also cloned into the luciferase reporter vectors to serve as non-specific controls. These constructs were transfected into HT-29 cells together with miRNA mimic (a chemically synthetic oligonucleotide that was designed to mimic endogenous mature miRNA molecules when transfected into cells). As shown in Figure 3B, compared with cells treated with the control vectors and the mutated sequences, luciferase activities were dramatically reduced in cells transfected with miR-17-5p, miR-19a, and miR-19b mimics, indicating that these sequences of EV71 genome are direct targets of these miRNAs. In contrast, miR-20a mimic did not affect luciferase activity. Moreover, miR-18a and miR-92a, which have no putative binding sites in EV71 genome, did not show any inhibitory effect on luciferase activity as expected. These results suggest that miR-17-5p and miR-19a/b may suppress EV71 replication by interacting with nt 3024-3038 and/or nt 2838-2862 of the viral genome. To confirm that miR-17-5p and miR-19a/b indeed suppressed EV71 replication, HT-29 and Caco-2 cells transfected with miR-17-5p or miR-19a/miR-19b mimic were infected with EV71 and the expression levels of viral proteins were determined by In-Cell-Western and Western blot analysis, respectively. These analyses revealed that EV71 VP1 was reduced in both miR-17-5p and miR-19b mimic-transfected cells (Figure 3C,D). Furthermore, the functional levels of miR-17-5p and miR-19b assembled in RISC were quantified by Ago2-IP and qRT-PCR and the levels of both miR-17-5p and miR-19b were found to be 3066-fold and 1800-fold enriched in the Ago2 IP fraction relative to the control. The level of U6, which is not expected to be associated with Ago2 in the RISC complex was only elevated 10.3-fold and 12-fold in the Ago2 IP, demonstrating the specificity of the Ago2 (Figure 3E). This result indicated that the transfection of miR-17-5p and miR-19b mimics significantly elevated the amount of the functional miRNAs. Collectively, our results were consistent with the fact that miR-17-5p and miR-19a/19b target the structural protein coding region of EV71 genome to inhibit viral replication.

### Altered DNA Methylation of the CpG Island in the miR-17-92 Cluster Promoter in EV71 Infection Cells

**FIGURE 4:**
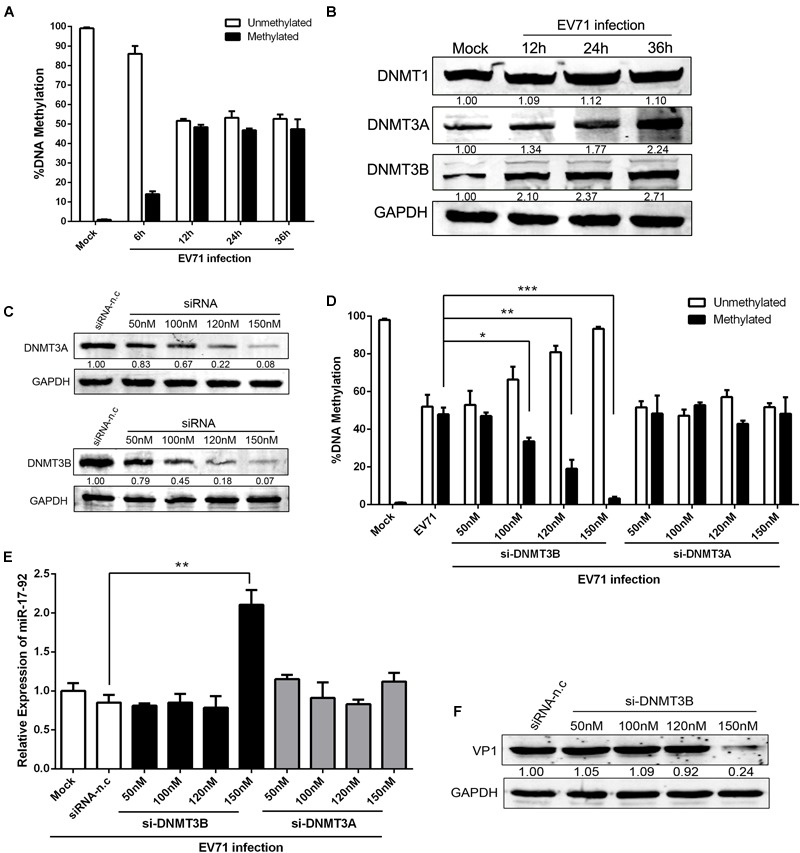
Altered DNA methylation of the CpG island in the miR-17-92 cluster promoter in EV71 infection cells. **(A)** DNA was isolated from mock and EV71-infected cells (*n* = 3). Data were presented as the average percent of unmethylated or methylated DNA of the miR-17-92 promoter. Statistical difference was determined by comparing the % unmethylated with % methylated for each group using QIAampDNA Mini Kit. **(B)** Western analysis of the expression levels of DNA methyltransferase (DNMT) enzymes 1, 3A, and 3B in EV71-infected HT-29 cells at the indicated time point. **(C)** HT-29 cells were transfected with DNMT3A- or 3B-specific siRNA for 48h and then detected by Western blot. The numbers represent the relative density of the band in comparison to the corresponding negative control normalized to GAPDH. Value of siRNA-n.c treatment is set at 1.00 (100%). **(D)** HT-29 cells were transfected with DNMT3A- or 3B-specific siRNA for 24 h and infected with EV71 at an MOI of 1. DNA was isolated and methylated DNA of the miR-17-92 promoter was determined by QIAampDNA Mini Kit. Shown is the percentage of DNA methylation, *n* = 4. **(E)** Pri-miR-17-92 cluster expression in cells transfected with si-DNMT3A- or 3B for 24 h and then infected with EV71 at an MOI of 1. **(F)** HT-29 cells was infected with EV71 (MOI = 1) prior to being transfected with si-DNMT3B for 24 h. EV71 VP1 protein expression level was determined via Western blot. Data were mean ± SEM of fold change and representative of at least three independent experiments (^∗^*P* < 0.05, ^∗∗^*P* < 0.01, ^∗∗∗^*P* < 0.001).

Epigenetic factors can also affect miRNA expression (Wang et al., 2017). Earlier study reported that more than 80% of the miR-17-92 promoter is consisted of a large CpG island (Dakhlallah et al., 2013). To investigate the mechanisms of the EV71-induced miR-17-92 downregulation, we analyzed the DNA methylation of this CpG island during EV71 infection with respect to miR-17-92 expression and showed that the DNA methylation of the miR-17-92 promoter was significantly elevated (∼50%) in EV71-infected cells as compared with the mock-infected cells (Figure 4A), suggesting that the DNA methylation of miR-17-92 promoter likely played a role in the downregulation of the miR-17-92 expression. To further illustrate the regulatory mechanisms of the miR-17-92 DNA methylation during EV71 infection, we next examined the expression of DNA methyltransferase (DNMT) enzymes 1, 3A and 3B. As shown in Figure 4B, the expressions of both DNMT3A and DNMT3B, but not DNMT1, were elevated in EV71-infected cells when compared with the mock-infected cells. This observation suggests that EV71 infection upregulated the expression of DNMT3A and 3B, which causes hypermethylation of the miR-17-92 promoter region, resulting in the downregulation of miR-17-92 expression.

To investigate the roles of DNMT3A/3B in the modulation of miR-17-92 expression, the cells were transfected with DNMT3A- or 3B-specific siRNA and infected with EV71 and the expression of miR-17-92 was determined. As shown in Figure 4C, the expression of both DNMT3A and 3B was reduced as the result of increasing specific siRNA dosage, resulting in more than 90% reduction in both DNMT3A and DNMT3B protein expression at 150 nM siRNA. Interestingly, the DNA promoter methylation of the miR-17-92 in EV71-infected cells was significantly reduced by the knockdown of DNMT3B but not DNMT3A (Figure 4D). Meanwhile, upon EV71 infection, the miR-17-92 expression was significantly affected by the siRNA-induced down-modulation of DNMT3B only at the concentration of 150 nM (Figure 4E). These results indicated that the miR-17-92 expression was tightly controlled by DNMT3B and miR-17-92 expression is highly sensitive to the methylation of its promoter since miR-17-92 expression above basal level was only detected when methylation was almost completely inhibited (at 150nM si-DNMT3B, Figure 4D,E). Consistent with the expression of miR-17-92, EV71 VP1 expression was significantly reduced only when the promoter methylation was fully inhibited in the presence of 150 nM of si-DNMT3B (Figure 4F).

We analyzed the gene promoters of top ten microRNAs which exhibited the most marked reduction upon EV71 infection by MethPrimer 2.0^1^ and found that no CpG islands were found in those promoter sequences except the miR-200c and miR-10a (Supplementary Figure S2). Both miR-200c and miR-10a contain much lower proportion of CpG relative to the length of their respective promoters than that of miR-17-92. With about 80% of the promoter sequence being CpG islands, miR-17-92 contains the highest potential sequence for methylation.

### miR-17-92 Was Reduced in Sera of EV71 Infected Patients

**FIGURE 5:**
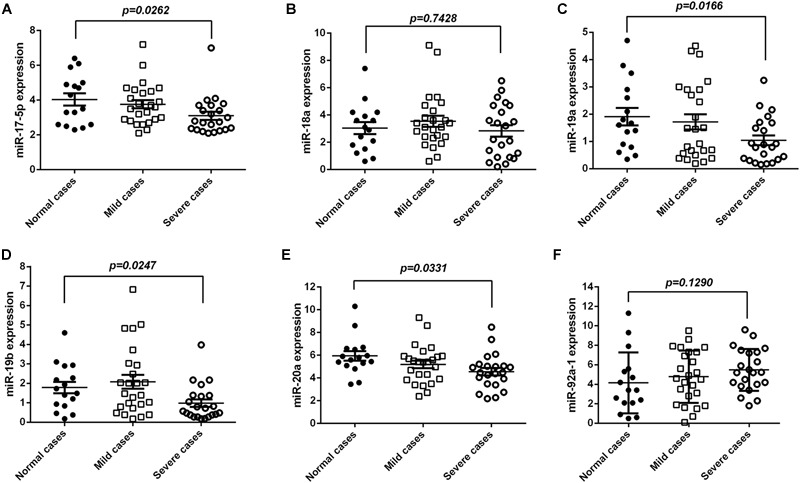
miR-17-92 was reduced in serum of EV71-infected patients. **(A–F)** The expression levels of mature miR-17-92 in serum specimens of EV71-infected patients by real-time quantitative PCR, and their correlation with disease severity. The EV71-infected patients were divided into a mildly ill group (sample = 25) and a severely ill group (sample = 22) as based on criteria of the Handbook for Treatment of HFMD.

To explore the biological relevance of miR-17-92 modulation in EV71 infection, we evaluated the mature miR-17-92 levels in serum specimens of EV71-infected patients. As shown in Figure 5A–F, the miR-17, miR-19a/b, and miR-20a were significantly reduced in severe cases (sample = 22) as compared with the normal cases (sample = 16) and mild cases (sample = 25). However, there were no significant differences for the miR-18a and miR-92a-1 between the severe and the normal cases. These results were consistent with our findings in the cell lines.

## Discussion

Increasing evidence has indicated miRNAs as critical effectors in the intricate networks of host-pathogen interactions which directly or indirectly affect virus replication and pathogenesis (Gottwein and Cullen, 2008; Skalsky and Cullen, 2010). In this study, to address whether cellular miRNAs are involved in the host response to EV71 infection, miRNA profiling of EV71-infected cells was performed, and the mature members of the miR-17-92 cluster were identified to be significantly reduced upon EV71 infection.

To investigate the potential biological roles of miR-17-92 cluster, chemically synthesized oligonucleotides were used to either overexpress or inhibit this cluster in EV71-infected cells. Overexpression of miR-17-92 significantly inhibited replication of EV71 replication, while inhibition of miR-17-92 facilitated viral replication. These results suggested that miR-17-92 cluster may play roles in the host’s antiviral activity and the virus overcomes this host resistance by downregulating the miR-17-92 expression. Likewise, evidence has shown that endogenous miR-17-92 cluster can directly interact with HBV genomes or indirectly effect on HIV-1, HCV or KHSV replication (Triboulet et al., 2007; Jung et al., 2013; Yang et al., 2013; Choi et al., 2015). Due to the similarity of RNA viral genomic structure with that of the host mRNA, it is possible that cellular miRNAs are capable of directly binding and degrading the RNA virus genome via the cell host machinery (Gottwein and Cullen, 2008). For instance, human miR-296-5p was found to suppress EV71 replication by targeting the viral genome (Zheng et al., 2013). hsa-miR-23b inhibited the replication of Fuyang.Anhui.P.R.C/17.08/1 isolate by targeting viral 3′UTR conserved sequence (Wen et al., 2013). Our earlier work showed that EV71 induced autophagy by suppressing miR-30a to facilitate its replication (Fu et al., 2015). Our present results illustrated that three members of the miR-17-92 cluster, miR-17-5p, miR-19a, and miR-19b, inhibited viral replication by directly binding to the VP1 region of the EV71 genome and interfering the structural protein synthesis.

Growing evidence has linked miRNA expression with DNA methylation process (Han et al., 2007). DNMTs are responsible for establishing and maintaining DNA methylation in mammalian cells and increased DNMT activity leads to the hypermethylation of cellular genes and miRNAs, which are generally associated with transcriptional repression and thereby contribute to gene regulation (Subramaniam et al., 2014; Hamidi et al., 2015). Here, we observed that the DNA methylation of the miR-17-92 promoter was significantly increased in EV71-infected cells, suggesting that downregulation of miR-17-92 was mediated through DNA methylation of its promoter. Previous report showed that the methylation of the miR-17-92 promoter was increased in pulmonary fibrosis and several miRNAs from the miR-17-92 cluster targeted DNMT-1 expression, resulting in a negative feedback loop (Dakhlallah et al., 2013). In the current study, the expression of DNMT-1 did not significantly change while DNMT3A and 3B protein expression increased upon EV71 infection, suggesting that DNMT3A or 3B contributed to the hypermethylation of the miR-17-92 promoter region. However, we can not rule out that DNMT-1 plays roles in the maintenance of miR-17-92 methylation since a kinetic study may be needed to detect changes of methylation, considering the high CpG content at the miR-17-92 promoter region. Importantly, inhibition of DNMT3B expression resulted in a significant inhibition of virus-induced methylation upregulation while inhibition of DNMT3A failed to inhibit the virus-induced upregulation of methylation, suggesting strongly that either DNMT3A-mediated methylation does not target at the miR-17-92 CpG region or DNMT3B can compensate the loss of DNMT3A activity but not *vis versa*. The expression of miR-17-92 appears to require complete inhibition of methylation of its promoter since miR-17-92 expression above basal level was detected only when less than 4% of the DNA was methylated (Figure 4D,E). We do not know yet the implication of the redundancy of DNMT3A and the biological roles of DNMT3A-catalyzed methylation during EV71 infection. It is possible that the DNMT3A-catalyzed methylation regulates other biological processes instead of miR-17-92. Previous research found that DNMT3A, the *de novo* DNA methyltransferase, was not specifically associated with microRNA in human cell lines and DNA methylation is not likely regulated by interaction between microRNA and DNMT3A in somatic cells (Park et al., 2010), which is consistent with our present findings. In addition, DNA methylation may regulate miRNA expression in an indirect manner by controlling the expression of transcription factors that regulate miRNA expression (Han et al., 2007; Hamidi et al., 2015). Importantly, it is suggested that EV71-induced hypermethylation of immune-related genes could block the generation of immune response against virus infection through down-regulating respective gene expression. For example, BST-2 expression, an important IFN-stimulated gene factors (ISGs), was inversely correlated with its DNA methylation status (Mahauad-Fernandez et al., 2015). Remarkably, DNA methylation also controlled gene transcription through interference with the ability of transcription factors to bind to DNA (Varley et al., 2013).

This is, to our best knowledge, the first report that miR-17-92 is regulated by methylation during viral infection. Considering that more than 80% of the miR-17-92 promoter is consisted of a large CpG island, it is not surprising to find high degrees of DNA methylation of this region. While our work has shed light on EV71-induced methylation of miR-17-92 promoter, details of the regulation and the viral factors or mechanisms that regulate the methylation require further investigation. The correlation of miR-17-5p and miR-19a/b with HFMD disease severity suggests that miR-17-92 may play important roles as an endogenous antiviral element.

## Materials and Methods

### Cell Cultures and Viruses

Human colorectal cell lines (HT-29 and Caco-2) were obtained from American Type Culture Collection (ATCC, United States) and maintained in Dulbecco’s modified Eagle’s medium (DMEM) containing 10% fetal bovine serum (FBS) in a 37°C humidified atmosphere of 5% CO_2_. The EV71 FY0805 strain was a kind gift from Dr. Bin Wu, Jiangsu Provincial Centers of Disease Control and propagated on the green monkey kidney cell line (Vero). Detailed protocols for preparing viral stocks can be found in our previous report (Fu et al., 2015).

### Western Blot

Western blot assay was performed as following: cells were lysed with RIPA buffer (Santa Cruz, CA, United States) and the extracted proteins were separated on 12% SDS-PAGE and then transferred onto PVDF membranes (Millipore, United States). The membranes were blocked for one hour at room temperature and then probed with primary antibodies at an appropriate dilution 4°C overnight, followed by incubation corresponding IRD Flour 680- or 800-labeled IgG secondary antibodies (Li-Cor Bioscience, United States). The membranes were scanned and quantified on Odyssey Infrared Imaging System. The primary antibodies against VP1 (Millipore, United States), DNMT-1 (Cell Signaling Technology), DNMT3A (Abcam), DNMT3B (Abcam) and GAPDH (Cell Signaling Technology) were purchased from respective companies.

### RT-PCR and Real-Time Polymerase Chain Reaction Analysis

Total RNA, including miRNA, was extracted from whole cell lysate using TRIzol reagent (Invitrogen). Reverse transcription and real-time polymerase chain reaction was performed as our previously described (Fu et al., 2017). Primers for microRNA and U6 (an internal control for the miRNA detection) were purchased from RiBoBio (Guangzhou, China). The sequences of PCR-amplified primer pairs were as follow:

EV71 genomic RNA: Forward: 5′-AGTGATATCCTGCAGACGGG-3′;
Reverse: 5′-ATAGCCCCAGACTGTTGTCC-3′;
pri-miR-17-92: Forward: 5′-ATAGTTGTTAGAGTTTGAGGTG-3′;
Reverse: 5′-GTACATTTAACAGTGGAAGTCG-3′.

The sequences of real-time PCR primer pairs were as follow:

EV71 genomic RNA: Forward: 5′-GCTCTATAGGAGATAGTGTGAGTAGGG-3′;
Reverse: 5′-ATGACTGCTCACCTGCGTGTT-3′.
GAPDH: Forward: 5′-TGCACCACCAACTGCTTAGC-3′;
Reverse: 5′-GGCATGGACTGTGGTCATGAG-3′.

### Ago2 Immunoprecipitation

HT-29 cells were transfected with miR-17-5p mimic or miR-19-3p. After 48 h, the cells were lysised in NP40 buffer supplemented with RNase inhibitor, protease inhibitor and phosphatase inhibitor (Sigma). The samples were then centrifuged for 15 min at 12,000 g at 4°C in a microcentrifuge. The supernatant was placed in a fresh tube kept on ice and subjected to Ago2 immunoprecipitation using the miRNA isolation kit for human Ago2 (Wako, Japan) according to the manufacturer recommendation. RNA derived from transfected cells following Ago2 immunoprecipitation were analyzed by real-time PCR.

### Luciferase Reporter Assay

An EV71 fragment (structure protein coding region; nt 1350-1803) containing presumptive miR-17-92 target sequences was amplified by PCR using the primers 5′-GGGGTACCAGTGATATCCTGCAGACGGG-3′ and 5′-GAAGATCTATAGCCCCAGACTGTTGTCC-3′ and a DNA template from HT-29 cells. The amplicon was cloned into the kpnI/BglII sites of the dual luciferase pmirGLO vector to generate pmirGLO-VG WT. The construct containing site region with mutant seed sequences of miR-17/20a and miR-19a/19b was also synthesized (pmirGLO-VG-mut). HT-29 cells were plated in 48-well plates and transfected with the pmirGLO-VG WT or pmirGLO-VG-mut, as well as the construct control and corresponding miRNA mimics. The dual luciferase assay was performed 48h after the final transfection according to the manufacturer’s instruction.

### In-Cell Western Assay

In-cell western assay was carried out as previously described (Lv et al., 2014).

### Transient Transfection of miRNA, siRNA and Plasmid

Cells grown were seeded onto 60mm plates with antibiotic-free medium and incubated until the confluence reached about 30∼50%. The cells were transfected using Lipofectamine 3000 (Life Technologies, United States) according to the manufacturer’s instructions. The final concentrations of miRNA mimics and their negative controls (RiBoBio, Guangzhou, China) were 100 nM. The efficiency of transfection was monitored by real-time PCR and western blot.

For siRNA transfection, the procedure was similar to that of miRNA transfection. After incubation with different dose of target siRNAs or scrambled siRNA (GenePharma, Shanghai, China) for 48 h, the cells were harvested and subjected to western blot analysis. The sequences of siRNA were as follow:

miR-17-92: 5′-AAGAGAACAUCACCUUGUA-3′(Triboulet et al., 2007)
DNMT3A: 5′-GGGTTGGACATCAYCTCCTGG-3′(Robert et al., 2003)
DNMT3B: 5′-CTCTGGGCACCTGTCATCTGG-3′(Bai et al., 2005)

For plasmid transfection, 80% confluent cells were transfected with a MSCV plasmid expressing intact miR-17-92 components using Lipofectamine 3000. 48 h later, the cells were infected with EV71 virus at MOI = 1 for indicated times.

### Small RNA Deep Sequencing Analysis

Total RNAs were extracted from HT-29 cells after infection with EV71 or mock for 24 h and analyzed by miRNA deep sequencing (ANOROAD, Beijing, China) as previously described (Fu et al., 2017).

### Examination of DNA Methylation Patterns

DNA was isolated using QIAampDNA Mini Kit (Qiagen, Valencia, CA, United States). DNA methylation was analyzed using the Methyl-Profiler DNA Methylation qPCR Primer Assays (Qiagen, Frederick, MD, United States) according to the instructions.

### Clinical Samples

Serum specimens were collected from pediatric patients with HFMD and other microbial infections as well as healthy children at Nanjing Children’s Hospital from April 2017 to June 2017. All serum samples prior to miRNA analysis were cryo-preserved at –80°C within 2 h following collection. Etiologic diagnosis of enterovirus infections in patients was confirmed by detection of EV71 using duplex real-time reverse-transcription PCR. This study was approved by the Ethics Committee of Jiangsu Provincial Center for Diseases Prevention and Control and written informed consent was obtained from parents or legal guardians of all children. All experiments were performed in accordance with relevant guidelines and regulations.

### Study Groups

Healthy and EV71-infected children were included in the study. EV71-infected patients were divided into a mildly ill group and a severely ill group following the criteria of the Handbook for Treatment of HFMD (2010) published by the NHFPC (Bartlett et al., 2015). Mild cases were diagnosed with HFMD, with or without fever while severe cases exhibited symptoms of CNS manifestations, as confirmed by the presence of clinical features including lethargy, irritability, headache, decreased reflex and muscle strength, myoclonus, ataxia, nystagmus, oculomotor palsy, and acute limb weakness, with or without neuroimaging.

## Author Contributions

YF performed experiments, analyzed the data, and wrote the initial manuscript. LZ, RZ, SX, and HW performed the experiments. YJ collected and maintained clinical samples and data. ZW conspired the study and revised the manuscript.

## Conflict of Interest Statement

The authors declare that the research was conducted in the absence of any commercial or financial relationships that could be construed as a potential conflict of interest.

## Footnotes

1 http://www.urogene.org/methprimer2
