# Supplementary material for: Enterovirus 71 Suppresses miR-17-92 Cluster Through Up-Regulating Methylation of the miRNA Promoter
Source: Front Microbiol. 2019 Mar 28;10:625. doi: 10.3389/fmicb.2019.00625 (PMC6447709; doi:10.3389/fmicb.2019.00625)

**Supplementary Information**

**Fig. S1：**Small RNA deep sequencing analysis on host cellular miRNAs in HT-29 cells infected with EV71 at a MOI of 1 for 24 h.


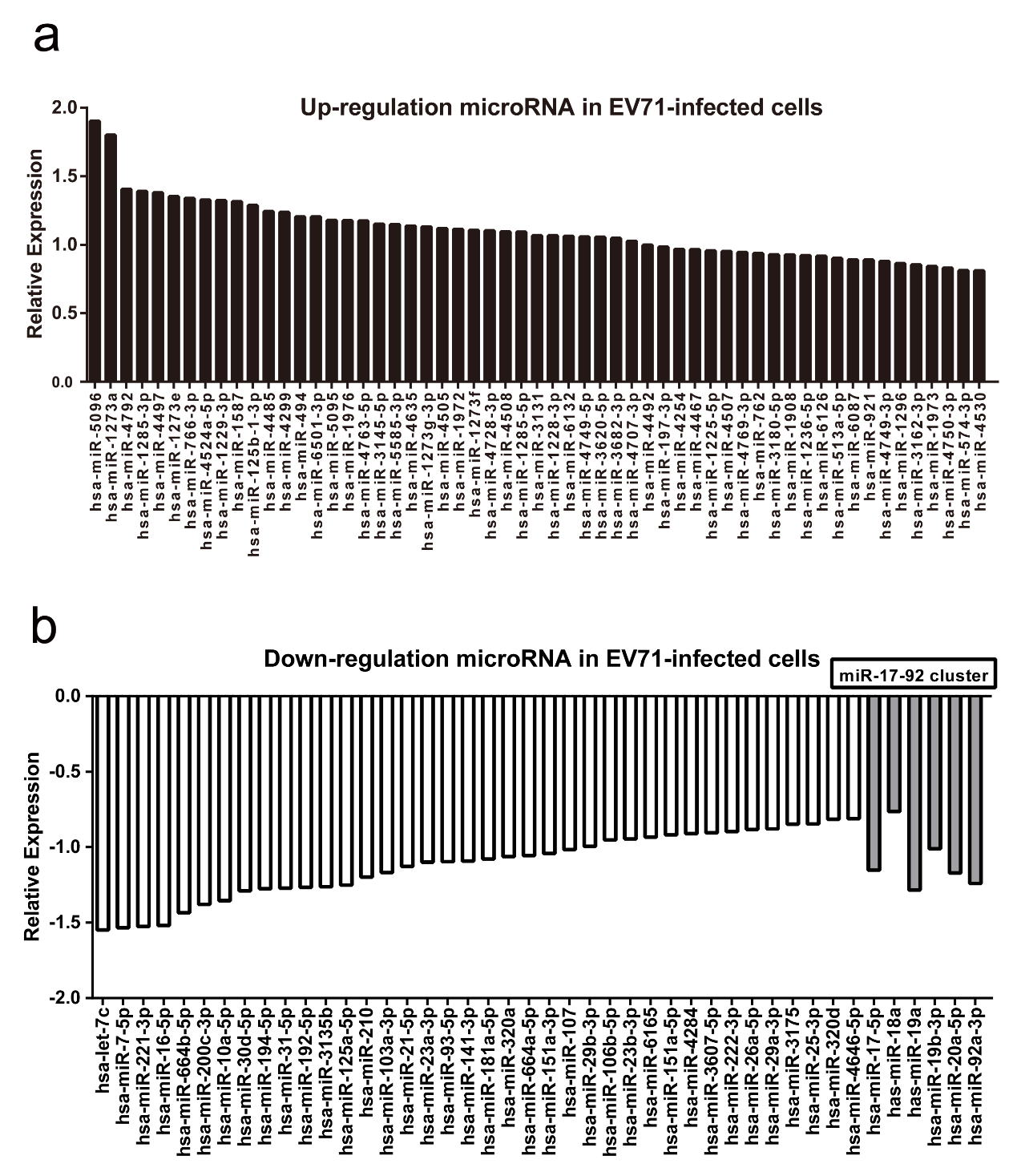


**Fig. S2:** The gene promoters of top ten microRNAs which exhibited the most marked reduction upon EV71 infection by MethPrimer 2.0.


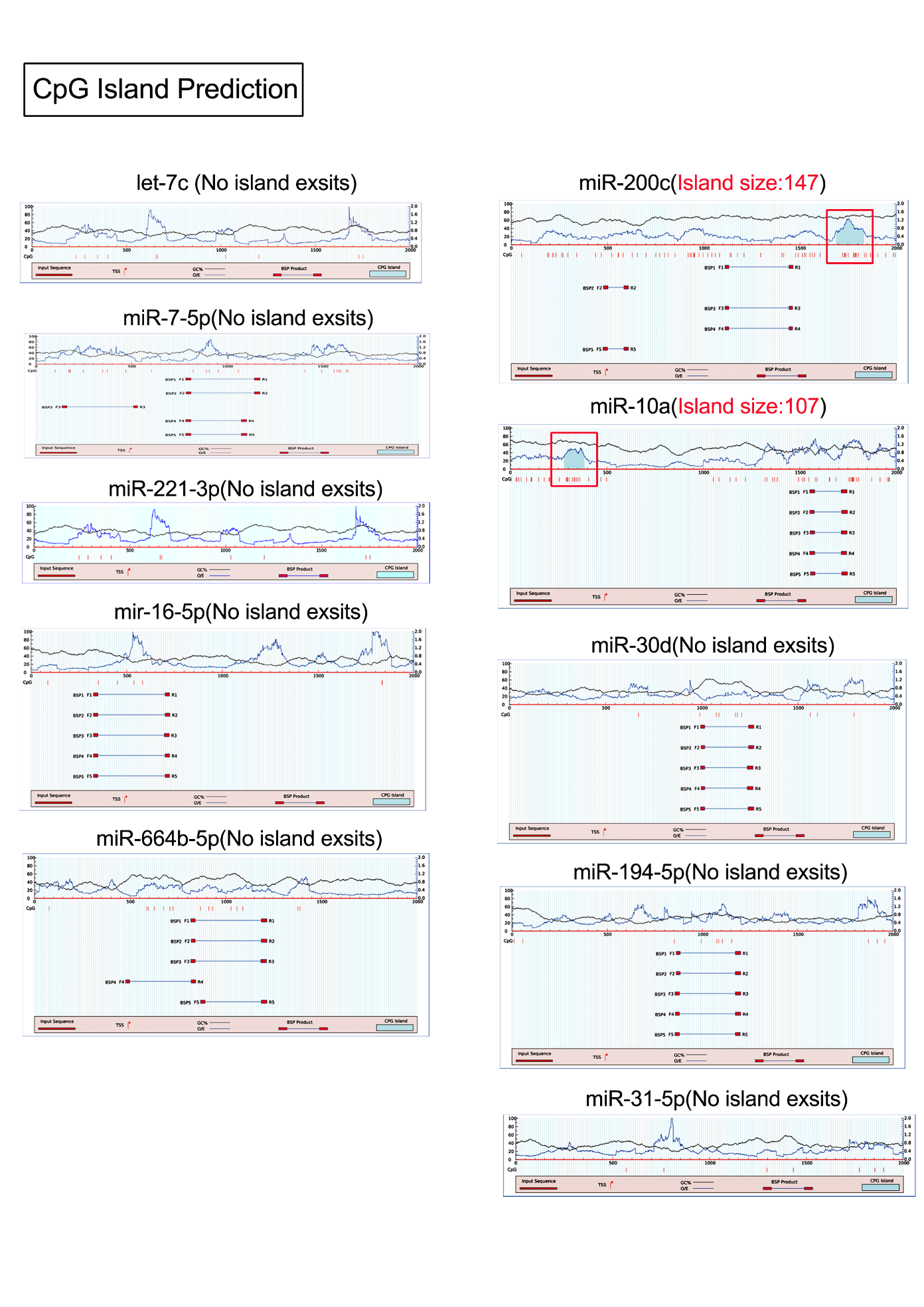

Supplement: Supplementary file 1 [file Data_Sheet_1.docx]
